# The Effects of tDCS Across the Spatial Frequencies and Orientations that Comprise the Contrast Sensitivity Function

**DOI:** 10.3389/fpsyg.2015.01784

**Published:** 2015-11-27

**Authors:** Bruno Richard, Aaron P. Johnson, Benjamin Thompson, Bruce C. Hansen

**Affiliations:** ^1^Department of Psychology, Concordia University, MontrealQC, Canada; ^2^Department of Psychology, University of YorkYork, UK; ^3^School of Optometry and Vision Science, University of Waterloo, WaterlooON, Canada; ^4^School of Optometry and Vision Science, The University of AucklandAuckland, New Zealand; ^5^Department of Psychology and Neuroscience Program, Colgate University, HamiltonNY, USA

**Keywords:** contrast sensitivity, transcranial Direct Current Stimulation (tDCS), spatial frequency, orientation, spatial vision

## Abstract

Transcranial Direct Current Stimulation (tDCS) has recently been employed in traditional psychophysical paradigms in an effort to measure direct manipulations on spatial frequency channel operations in the early visual system. However, the effects of tDCS on contrast sensitivity have only been measured at a single spatial frequency and orientation. Since contrast sensitivity is known to depend on spatial frequency and orientation, we ask how the effects of anodal and cathodal tDCS may vary according to these dimensions. We measured contrast sensitivity with sinusoidal gratings at four different spatial frequencies (0.5, 4, 8, and 12 cycles/°), two orientations (45° Oblique and Horizontal), and for two stimulus size conditions [fixed size (3°) and fixed period (1.5 cycles)]. Only contrast sensitivity measured with a 45° oblique grating with a spatial frequency of 8 cycles/° (period = 1.5 cycles) demonstrated clear polarity specific effects of tDCS, whereby cathodal tDCS increased and anodal tDCS decreased contrast sensitivity. Overall, effects of tDCS were largest for oblique stimuli presented at high spatial frequencies (i.e., 8 and 12 cycles/°), and were small or absent at lower spatial frequencies, other orientations and stimulus size. Thus, the impact of tDCS on contrast sensitivity, and therefore on spatial frequency channel operations, is opposite in direction to other behavioral effects of tDCS, and only measurable in stimuli that generally elicit lower contrast sensitivity (e.g., oblique gratings with period of 1.5 cycles at spatial frequencies above the peak of the contrast sensitivity function).

## Introduction

Neuro-stimulation techniques have recently been combined with traditional psychophysical paradigms in an effort to obtain a measure of direct manipulation on spatial frequency channel operations in the early visual system (review: Antal et al., 2006). One technique that is gaining popularity due to its affordability and simplicity is transcranial Direct Current Stimulation (tDCS), a non-invasive brain stimulation technique that transiently modulates excitation and inhibition in the human brain via alterations in the membrane potential of neurons (Antal et al., 2001, 2006; Nitsche et al., 2008; Stagg et al., 2009; Stagg and Nitsche, 2011). The technique involves a stimulating device that delivers a mild direct current (DC) between two electrodes (anode and cathode) placed on the scalp of an observer, which creates a resistive DC circuit that induces a mild intra-cerebral electrical current from the anode where current enters cortex, to the cathode where current exits the cortex. The direction of current flow determines the effect of tDCS. Specifically, anodal stimulation (a-tDCS) generates a sub-threshold depolarization, while cathodal (c-tDCS) stimulation hyperpolarizes the membrane potential of neurons (Radman et al., 2009; Reato et al., 2010; Paulus, 2011; Stagg and Nitsche, 2011; Pellicciari et al., 2013; Rahman et al., 2013). Polarity specific behavioral effects of tDCS are well established in motor cortex (e.g., Jacobson et al., 2012). However, in primary visual cortex, it is typical to find either facilitatory or inhibitory effects due to a-tDCS or c-tDCS, but not both. Also, the polarity specific facilitation and inhibitory effects of tDCS may be opposite to those reported in motor cortex (Antal et al., 2001; Accornero et al., 2007; Lang et al., 2007; Chaieb et al., 2008; Spiegel et al., 2012; Peters et al., 2013; Pirulli et al., 2014). Part of the variability in tDCS effects for different cortical loci can be attributed to structural (e.g., cell type and morphology and the direction of current flow in relation to the somatodendritic axis), or functional differences between stimulated areas (Rushton, 1927; Ward and Weiskrantz, 1969; Shipp, 2005; Radman et al., 2009; Reato et al., 2010; Bikson et al., 2013). Given that the visual cortex is both structurally and functionally different from motor cortex, it should come as no surprise that the effects of tDCS over the visual cortex are less clear.

The application of a-tDCS over primary visual cortex has been shown to enhance contrast sensitivity in amblyopic persons (Spiegel et al., 2013) at spatial frequencies above the peak of the contrast sensitivity function (CSF) and near the peak of the CSF (Kraft et al., 2010) while inhibitory effects of c-tDCS (Antal et al., 2001; Chaieb et al., 2008) on contrast sensitivity have been found for spatial frequencies above the peak of the CSF. However, all previous studies of tDCS on contrast sensitivity presented a single spatial frequency to observers, and thus, the effect of tDCS on the shape of the CSF (Campbell et al., 1966; Graham, 1989; Peli et al., 1993), which involves multiple spatial frequencies, is currently unknown^1^. Furthermore, the influence of stimulus orientation on tDCS induced changes in contrast sensitivity has not been investigated.

The goal of the current study was to assess how the effects of tDCS vary according to the stimulus dimensions (spatial frequency and orientation) used to measure contrast sensitivity. Given the known functional organization of the early visual system, and the properties of the DC circuit generated by tDCS, certain predictions as to the interaction of tDCS and stimulus dimension can be made. First, the effects of tDCS on contrast sensitivity should be greatest at higher spatial frequencies, and diminish with decreasing spatial frequency. This is because tDCS exerts its greatest effect at cortical sites closet to the skull (Miranda et al., 2006, 2013; Rahman et al., 2013) and V1 neurons at the occipital pole (close to the skull) have higher preferred spatial frequencies than those located deeper within the calcarine sulcus (Tootell et al., 1981, 1988; De Valois et al., 1982; Foster et al., 1985; Engel et al., 1997; Horton, 2006; Henriksson et al., 2008; Yu et al., 2010). Cells further from the occipital pole have receptive fields located peripherally in the visual field, which means that stimuli presented further than 2° eccentricity from fovea may not be affected as strongly by tDCS than stimuli presented in the central visual field (Kraft et al., 2010; but see Costa et al., 2015 for a contrasting view). Stimulus orientation may also influence the effect of tDCS on contrast sensitivity. Contrast sensitivity to oblique gratings is lower than that to horizontal gratings (the “Oblique Effect”; Campbell et al., 1966; Appelle, 1972; Essock, 1980). Therefore, contrast sensitivity to oblique gratings may be more susceptible to the facilitatory effects of a-tDCS whereas horizontal gratings may be more susceptible to the inhibitory effects of c-tDCS. This, in essence, should decrease the magnitude of the “Oblique Effect”. Thus, we measured changes in contrast sensitivity from a non-stimulation baseline under both a-tDCS and c-tDCS to gratings of four different spatial frequencies that spanned the CSF (0.5, 4, 8, and 12 cycles/°) and two stimulus orientations (45° oblique or Horizontal).

## Materials and Methods

### Participants

Twenty-six undergraduate students participated at baseline, out of which 20 continued onto the tDCS portion of this study. All observers but two were naïve to the goals of the experiment. Observers were prevented from moving onto the tDCS sessions when their contrast detection thresholds measured just prior to the application of tDCS exceeded 2 SDs of their average thresholds measured at baseline. Participants that continued onto the tDCS sessions were separated into two groups; 10 (*N*_female_ = 7, *M*_age_ = 20.2) participants were presented with oblique gratings while the other 10 (*N*_female_ = 5, *M*_age_ = 20.5) saw horizontal gratings. Two of the participants in the oblique orientation group completed the experiment at Concordia University (Montreal, QC, Canada), while data for all other participants in this study were collected at Colgate University (Hamilton, NY, USA). All participants had normal, or corrected-to-normal visual acuity (Snellen cutoff = 20/25) and no astigmatism. Written informed consent was obtained from all participants and all were treated in accordance to the Tri-Council Policy Statement: Ethical Conduct for Research Involving Humans (Medical Research Council of Canada, 2003) and the ethical standards of the Federal Code of Regulations Title 45 (Public Welfare) and Department of Health and Humans Services, Part 46 (Protection of Human Subjects). All participants were compensated financially for their time.

### Apparatus

All stimuli were presented on 22.5″ Viewsonic (G225fB) monitors driven by a dual core Intel^®^ Xeon^®^ processor (1.60 GHz x2) equipped with 4GB RAM and a 256MB PCIe x16 ATI FireGL V7200 dual DVI/VGA graphics card with 8-bit grayscale resolution at Colgate University and an Apple Mac Pro (2x 2.66 GHz processor) equipped with 8GB of RAM and a 1GB PCIe x16 ATI Radeon HD 5770 Graphics card with 8-bit grayscale resolution. The color management settings for the graphics card (i.e., 3D display settings) were adjusted such that the luminance “gain” of the green gun was twice that of the red gun, which was set to twice that of the blue gun. A bit-stealing algorithm (Tyler, 1997; Bex et al., 2007) was employed to yield 10.8 bits of luminance (i.e., grayscale) resolution (i.e., 1785 unique levels) distributed evenly across a 0–255 scale. Stimuli were displayed using a linearized look-up table, generated by calibrating with a Color-Vision Spyder3 Pro sensor. Maximum luminance output of both display monitors was 100 cd/m^2^ (50 cd/m^2^ mean luminance after calibration). The frame refresh rate was set to 85 Hz (100 Hz at Concordia), and the resolution was set to 1600 × 1200 pixels (1024 × 768 pixels at Concordia). Single pixels subtended 0.0134° (0.0381° at Concordia) of visual angle, i.e., 0.80 arc min. (2.28 arc min at Concordia) as viewed from 1.0 m. Head position was maintained with a chin rest. Participants viewed the display monitor from 2 m in a dark room through an aperture (16° of visual angle in diameter) of a large black circular mask that was fit to the monitor bezel in order to obscure any monitor or room orientation cues.

Transcranial Direct Current was generated with a 9V battery driven direct current stimulator (Chattanooga Ionto, USA) and delivered via a pair of carbon-rubber electrodes (The Magstim Company Ltd., UK). The electrodes were encased in potassium chloride soaked Spontex sponge pockets (The Magstim Company Ltd., UK). The size of the stimulating electrode was 6 cm × 8 cm, and the size of the reference electrode was 12 cm × 8 cm. The larger size of the reference electrode renders it inert due to low current density (Nitsche et al., 2007; Spiegel et al., 2012). Both electrodes were held in place with four Magstim rubber headbands (The Magstim Company Ltd., UK), applied in a manner that maximized complete electrode sponge surface contact over the targeted scalp regions.

### Stimuli

Stimuli consisted of foveally presented sinusoidal gratings generated at one of two orientations: either oblique (45°) or horizontal (90°). All gratings were windowed by a 2D Gaussian, which ramped down the contrast to mean luminance. Stimulus spatial frequency was 0.5, 4, 8, or 12 cycles/°, with a period of 1.5 cycles (fixed period condition). The electrical field generated by tDCS is prominently focused onto the surface of the visual cortex, which limits the spatial extent of the visual field modulated by tDCS to the central 1–2° of the visual field (Kraft et al., 2010)^2^. As the effects of tDCS change as both a function of spatial frequency and stimulus area, we added a second stimulus condition and measured contrast sensitivity with a fixed stimulus size (3°), and adjusted the period of the stimulus with spatial frequency (fixed stimulus size condition). All stimuli were surrounded by a low contrast ring (Michelson Contrast = 10%) 1 pixel in size, 0.78° away from the border of the grating, and paired with a low frequency tone; both served to minimize participant doubt as to the location and/or presence of the stimulus on the screen. Stimulus contrast was expressed as Michelson contrast = [(L_max_ - L_min_)/(L_max_ + L_min_)] scaled to have zero mean and then normalized to 1.0.

### Psychophysical Procedure

The within-subject stimulus conditions for this experiment consisted of four spatial frequencies (0.5, 4, 8, and 12 cycles/°), and two period conditions (fixed period and fixed size). Observers were grouped according to the stimulus orientation (45° oblique or horizontal). The psychophysical procedure for both the training and test phases were identical. The stimulus presentation consisted of a 2-Interval Force Choice (2-IFC) procedure where participants had to indicate the interval, either the first or the second, which contained the target. Target contrast was controlled by a 2-up, 1-down staircase setup and controlled by the *PAL_AMUD_setupUD* and the *PAL_AMUD_updateUD* functions from the Palamedes toolbox for MATLAB (Prins and Kingdom, 2009; Kingdom and Prins, 2010). Threshold was approached from above with a target contrast step size of 0.05% Michelson contrast. Each staircase ran until 12 reversals were observed and the averaged target contrast value of the last five reversals was used as an estimate of target contrast threshold (70.71% correct on the psychometric function).

**FIGURE 1:**
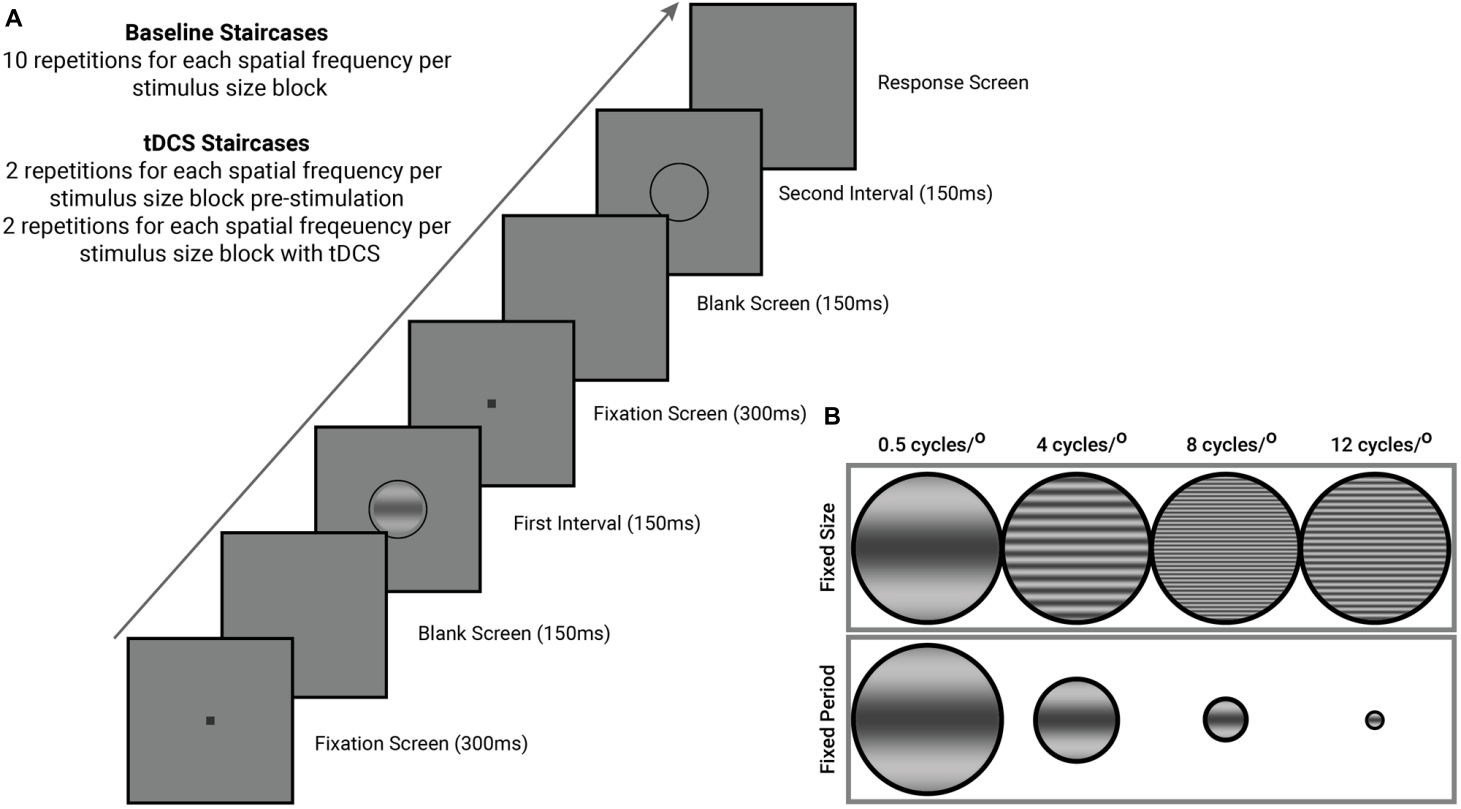
**General psychophysical procedures completed by all observers in this study. (A)** Stimulus presentation sequence (see text for details). **(B)** Contrast sensitivity was measured for both stimuli of a fixed size and fixed period, at four different spatial frequencies (0.5, 4, 8, and 12 cycles/°). Groups (*n* = 10 per group) were split according to stimulus orientation (45° oblique, and horizontal). Stimuli in the fixed period condition do not represent the actual change in size of our stimuli during the staircase, and are a graphical representation of the different stimulus dimensions used in this study. Stimuli of a fixed size subtended 3° of visual angle while stimuli of a fixed period had a period of 1.5 cycles.

All staircases completed by observers began with an instruction screen that informed them of the spatial frequency and size condition of the stimulus (orientation never changed within observers). Each trial began with a black fixation dot (0.1°) presented at the center of the screen. The fixation dot served both to remind the observer a stimulus will appear shortly and the location of said stimulus. The fixation screen (300 ms) was followed by a blank screen (150 ms) set to mean luminance, followed by the first stimulus interval (onset followed a square-wave function) presented for 150 ms. This sequence was repeated for the second stimulus interval (see **Figure 1**). One interval contained the stimulus, surrounded by a low-contrast ring, while the other interval contained only a low-contrast ring. Participants indicated, via keyboard press, the interval that they believed contained the target. The duration of the response interval was unlimited, and participants received no feedback on their accuracy.

Each spatial frequency by stimulus size block was repeated 10 times by observers in the baseline portion of the study (total of 80 staircase blocks), which approximately took 5 h to complete over multiple 1-h sessions completed on different days (approximately five sessions over 2 weeks). All staircase blocks were randomly interleaved for each observer, and only the final eight stimulus blocks were stored for data analysis. The contrast sensitivity of observers across each sequential measurement for all spatial frequency and stimulus size conditions is shown in Figure A1 (see Supplementary Material A), separated by orientation group. The 20 observers (10 per orientation group) that continued onto the tDCS portion of this study showed no statistically significant increment or decrement in contrast sensitivity across the final eight stimulus blocks completed during baseline (the slope of the line of best fit across all eight stimulus blocks was not statistically different from 0, all *p*s > 0.05). This is consistent with other studies that have shown either small (Sowden et al., 2002; Li et al., 2009), or no change in the CSF over sequential measurements in healthy adults (Dorais and Sagi, 1997; Adini et al., 2002, 2004; Maehara and Goryo, 2007).

### tDCS Procedure

Transcranial Direct Current Stimulation is known to be a safe neuro-stimulation technique with no long lasting negative side effects, it is nevertheless important to limit the duration of stimulation to no more than 30–35 min (Nitsche et al., 2003b; Poreisz et al., 2007; Bikson et al., 2009; Russo et al., 2013; Fertonani et al., 2015). In order to meet this time restriction, the number of repetitions for each spatial frequency by stimulus size block was set to two. The total number of staircases completed by observers while receiving tDCS was 16 (four spatial frequencies by two stimulus size conditions by two repetitions). Prior to receiving either a-tDCS or c-tDCS, participants completed two staircases for each spatial frequency by stimulus size blocks, which were combined with the eight stimulus blocks from the baseline portion of this study and used as a pre-stimulation baseline (see Supplementary Material A, Figure A2). If contrast detection thresholds exceed their average baseline thresholds by at least 2 SDs, participants were asked to repeat the pre-stimulation baseline measurements. If thresholds following the repetition remained 2 SDs away from average thresholds, participants were excused from the study.

Immediately following baseline measurements, participants repeated the 16 staircases while receiving tDCS (time to complete: *M* = 21.05 min, *SD* = 2.74). All observers completed two stimulation sessions (anodal and cathodal, counterbalanced across participants) with no less than 48 hours between sessions. As both a-tDCS and c-tDCS have been shown to produce differential effects on contrast detection performance (see Antal et al., 2001; Kraft et al., 2010; Jacobson et al., 2012; Spiegel et al., 2012), we used both stimulation conditions to serve as a control of the other. Specifically, we prioritize any relative effects whereby tDCS polarity differentially modulated contrast sensitivity for a particular stimulus dimension within our observers. This allowed us to avoid certain confounds that have been associated with sham in neurostimulation designs (for review: Duecker and Sack, 2015). Specifically, while observers are typically unable to differentiate between a-tDCS and c-tDCS, they have been shown to easily detect the sham condition, which may alter their response pattern and thus, serves as a poor control for neurostimulation (Minhas et al., 2011; Kessler et al., 2012; O’Connell et al., 2012).

Injecting current was set to 2 mA, which yielded a stimulation current density of 0.042 mA/cm^2^ over primary visual cortex. The stimulation and reference electrode were positioned over Oz and Cz, respectively, in accordance with the 10–20 EEG system (Chatrian et al., 1985; Antal et al., 2004a). The current was initially ramped up, over a period of 30 s and participants waited for a minute once the current ramped-up so the experimenter could verify comfort levels. When participants completed the 16 staircases, the current was ramped back down to zero over a period of 30 s. Once the experimental session was completed, participants completed a post-stimulation checklist to verify for any minor side-effects (Nitsche et al., 2008) – none were reported.

### Statistical Analyses

Contrast detection thresholds (*c*_threshold_) were transformed to dB sensitivity units Contrast Sensitivity _db_ = 20log _10_(1/c_threshold_) prior to analyses. The first statistical analysis conducted for all stimulus block conditions (stimulus orientation by stimulus period condition), was a 2 (tDCS polarity) × 4 (spatial frequency) repeated measures ANOVA on the difference contrast sensitivity values (stimulation – pre-stimulation), which tested for any spatial frequency dependent or polarity specific effect of tDCS on contrast sensitivity. All statistically significant interactions were followed by simple effect analyses. ANOVA output tables for all analyses are reported in Supplementary Material B.

Additionally, this study was designed to serve as a potential reference for future experiments that aim to use contrast sensitivity as a dependent measure of tDCS effects, but direct comparison between studies is complicated when only *p*-values are reported (see Kline, 2004 - Chapter 3 – for an in-depth description of the issues associated with null-hypothesis significance testing and *p*-values). Thus, we report an additional effect size analysis, which measured the magnitude of effects both at the group level (Hedge’s g) and at the case level (e.g., Left Tail Ratios, LTRs). The advantage of effect size measures is that their expected values are independent of sample size and thus they simplify the interpretation of results (particularly in regards to comparisons with other studies) and promote replication. The magnitude of an effect size should be interpreted in context to the relevant literature (Cohen, 1988). Thus, we interpret effect size magnitude according to the meta-analysis findings of Jacobson et al. (2012). They reported average effect sizes (g) of approximately 1.11 (CI [0.53 – 2.04]) of a-tDCS and 0.56 (CI [0.04 – 1.22]) of c-tDCS in cognitive studies (i.e., studies that measured the impact of tDCS on language, attention/perception, executive function, and memory). Any effect size that exceeds the average effect of either a-tDCS or c-tDCS is considered large, while effect sizes below the average values are moderate or small. LTRs are a case level analysis designed to assess the relative proportion of contrast sensitivity measurements recorded during stimulation to those of pre-stimulation in the left-tail of the combined distribution (see Supplementary Material B). Under assumptions of normality, homogeneity of variance, and large and equal group sizes, case-level proportions are functions of the magnitude of effect size at the group-level (Kline, 2004). However, when these assumptions are not met, group-level and case-level analyses will both offer separate information on the obtained effects. Given that the current that enters cortex with tDCS is several orders of magnitude less than what is required to elicit action potentials, any influence of tDCS on psychophysical performance will be relatively small, and may only be large enough in a sub-group of our sample (see Spiegel et al., 2013). Thus, the combination of group-level and case-level analyses offer a thorough descriptive approach of the data by quantifying effects in both central tendency and spread of the distribution of contrast sensitivity values. LTRs are calculated with the largest proportion as the numerator (regardless of time-point affiliation); values marked by an asterisk (^∗^) indicate that the pre-stimulation contrast sensitivity values were over-represented in the left tail of the combined distribution. Finally, interval estimates reported for Hedge’s g effect size measures are exact 95% confidence intervals calculate from the non-central t distribution (see Supplementary Material B; Cumming and Finch, 2001; Kline, 2004). Interval estimates for $\eta_{p}^{2}$ variance accounted for effect sizes are not reported, as their distribution in correlated designs are complex and do not follow a central nor a non-central distribution (Cumming and Finch, 2001; Kline, 2004).

## Results

**FIGURE 2:**
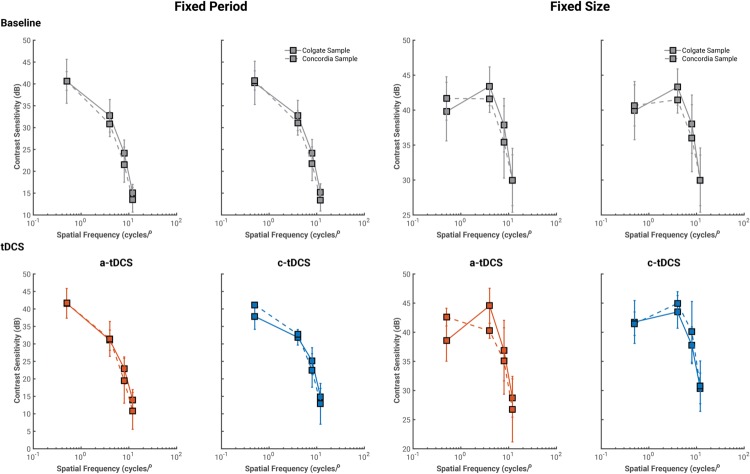
**Average contrast sensitivity values collected from the Colgate University (solid lines) and Concordia University (dashed lines) at baseline (gray) and tDCS sessions.** For all conditions, contrast sensitivity values from both samples overlapped significantly and thus, were averaged for all subsequent analyses.

Two observers in the oblique condition completed the study at Concordia University, and thus, we first verified that their contrast sensitivity values were similar to those of the Colgate University sample (see **Figure 2**). We report U1 (see Supplementary Material B; Cohen, 1988), a statistic of overlap with range [0–1]: values of 0 indicate complete overlap between both samples, while values of 1 indicate no overlap whatsoever. At baseline, there was significant overlap between contrast sensitivity measures collected at both testing facilities (U1 never exceeded 0.27). Both a-tDCS and c-tDCS measures showed similar results to those of baseline, except for the fixed size stimuli with spatial frequency of 4 cycles/°, U1 = 0.87. This shows little overlap between scores from the Colgate and Concordia samples. However, given that contrast sensitivity values were discrepant for a single stimulus condition block, we average contrast sensitivity values collected at both testing locations for all subsequent analyses.

### Fixed Period Oblique and Horizontal Stimuli

**FIGURE 3:**
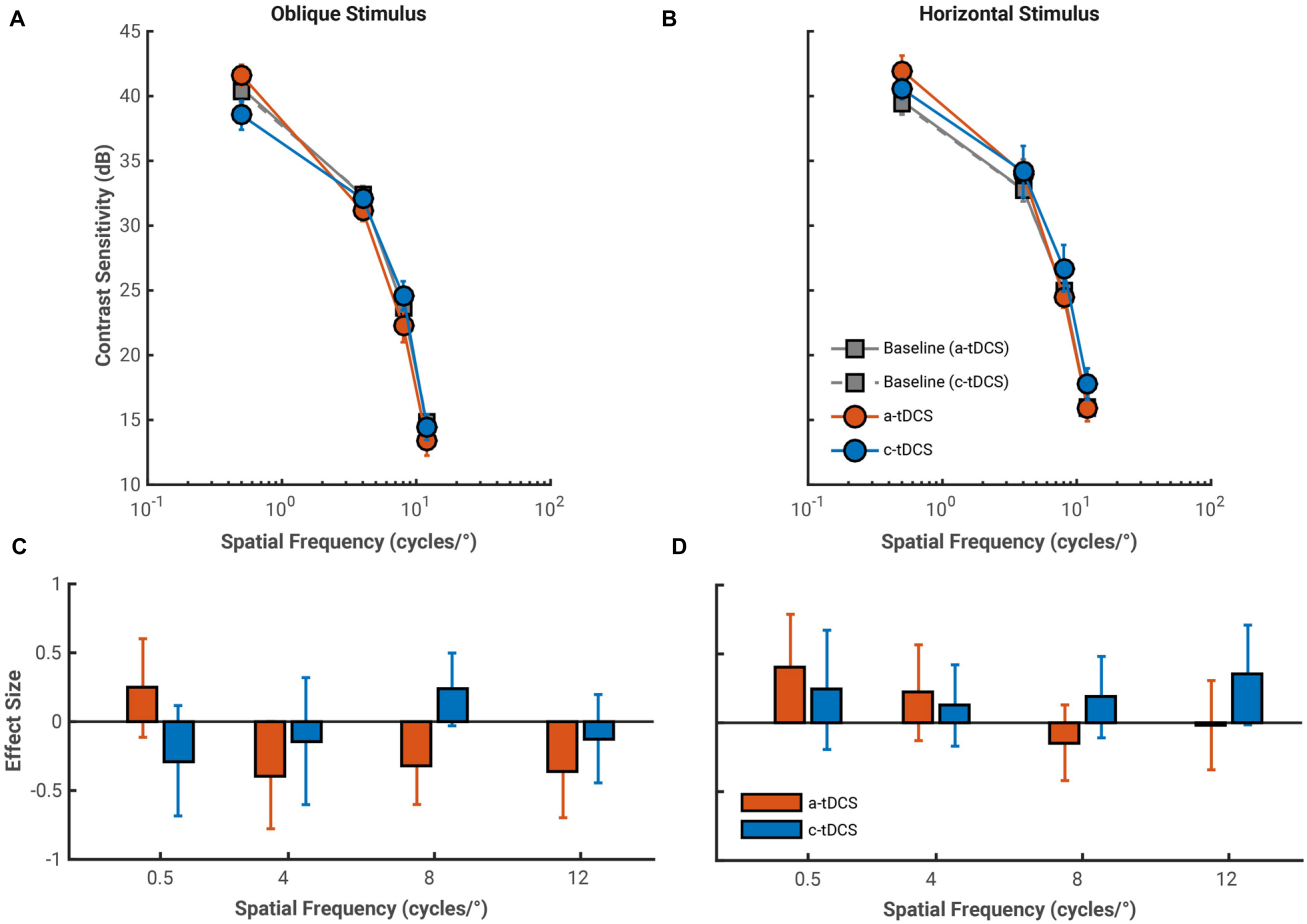
**Average pre-stimulation (gray) and stimulation contrast sensitivity functions (CSFs) for both a-tDCS (red) and c-tDCS (blue) measured with the oblique **(A)** and horizontal **(B)** fixed period gratings (at spatial frequencies of 0.5, 4, 8, and 12 cycles/°).** Contrast sensitivity is presented in decibels (dB). Error bars represent the standard error of the mean difference calculated across observers. **(C,D)** The effect sizes of the mean difference contrast sensitivity measured at stimulation and at pre-stimulation for oblique and horizontal conditions, respectively. For oblique gratings, contrast sensitivity measured at 8 cycles/° showed a polarity specific effect of tDCS, whereby a-tDCS decreased and c-tDCS increased contrast sensitivity. Error bars represent the exact 95% confidence interval of the effect size. We used error bar overlap to assess the magnitude of tDCS effects on contrast sensitivity. Thus, error bars that do not contain 0 and do not overlap with changes in contrast sensitivity with the other tDCS polarity are considered “significant”.

The average effects of both a-tDCS and c-tDCS on fixed period oblique and horizontal gratings are shown in **Figure 3**. Contrast sensitivity measured with oblique fixed period gratings showed a statistically significant interaction between tDCS polarity and spatial frequency, *F*(3,27) = 8.10, *p* < 0.001, $\eta_{p}^{2}$ = 0.474, which stemmed from a contrast sensitivity decrease under a-tDCS and increase under c-tDCS at a spatial frequency of 8 cycles/°, *F*(1,9) = 20.79, *p* < 0.001, $\eta_{p}^{2}$ = 0.698. There was no statistically significant interaction between spatial frequency and tDCS type on contrast sensitivity measured with horizontal fixed period gratings, *F*(3,27) = 1.97, *p* = 0.585, $\eta_{p}^{2}$ = 0.179.

The effect size analysis also showed the polarity specific effect of tDCS on contrast sensitivity measured to an 8 cycles/° oblique grating (**Figure 3C**). Contrast sensitivity decreased by a third of a standard deviation under a-tDCS (8 cycles/°: *g* = –0.32, 95% CI [–0.60 –0.03]) while it increased by a quarter of a standard deviation under c-tDCS (g = 0.24, 95% CI [–0.03 0.50]). Additionally, we found a-tDCS to decrease contrast sensitivity by a similar amount at spatial frequencies of 4 cycles/° (*g* = –0.40, 95% CI [–0.78 –0.03]) and 12 cycles/° (*g* = –0.36, 95% CI [–0.70 –0.01]). At the group level, a-tDCS induced decreases in contrast sensitivity remained stable across spatial frequency, but at the case-level, we found that observers were progressively more likely to have contrast sensitivity values 1 SD below the grand mean than pre-stimulation contrast sensitivity values as spatial frequency increased. This would suggest that these decrements in contrast sensitivity under a-tDCS are accentuated with spatial frequency (see **Table 1**). Thus, the effects of a-tDCS may be spatial frequency dependent, and increase in magnitude in accordance with an increase in spatial frequency.

**Table 1:** Left-Tail Ratios of contrast sensitivity measures in the fixed stimulus period condition.

		Spatial frequency (cycles/°)			
Stimulus dimensions		0.5	4	8	12
**45° Oblique**					
	a-tDCS	2.50^∗^	4.34	12.67	124.19
	c-tDCS	6.47	1.02^∗^	1.66	16.48
**Horizontal**					
	a-tDCS	1.74^∗^	1.34^∗^	1.23	3.39
	c-tDCS	2.56^∗^	24.80	7.52	1.70

*Values marked with an asterisk (^∗^) are ratios with the proportion of scores from the pre-stimulation distribution as the numerator.*

The effects of a-tDCS and c-tDCS on horizontal fixed period gratings were small in comparison to those of its oblique counterpart. We did find a moderate increment in contrast sensitivity under c-tDCS at a spatial frequency of 12 cycles/° (*g* = 0.35, 95% CI [–0.02 0.71]). This effect may be spatial frequency dependent, as the both the effect size and LTRs (see **Table 1**) showed that the benefit of c-tDCS on contrast sensitivity increased with spatial frequency: from 4 cycles/° (*g* = 0.13, 95% CI [–0.17 0.42]) and 8 cycles/° (*g* = 0.19, 95% CI [–0.11 0.48]), which reached significance at 12 cycles/°. Thus, the results of the fixed period condition show that the effects of a-tDCS may be most pronounced on oblique gratings while those of c-tDCS on horizontal gratings, both for spatial frequencies above the peak of the CSF.

### Fixed Size Oblique and Horizontal Stimuli

**FIGURE 4:**
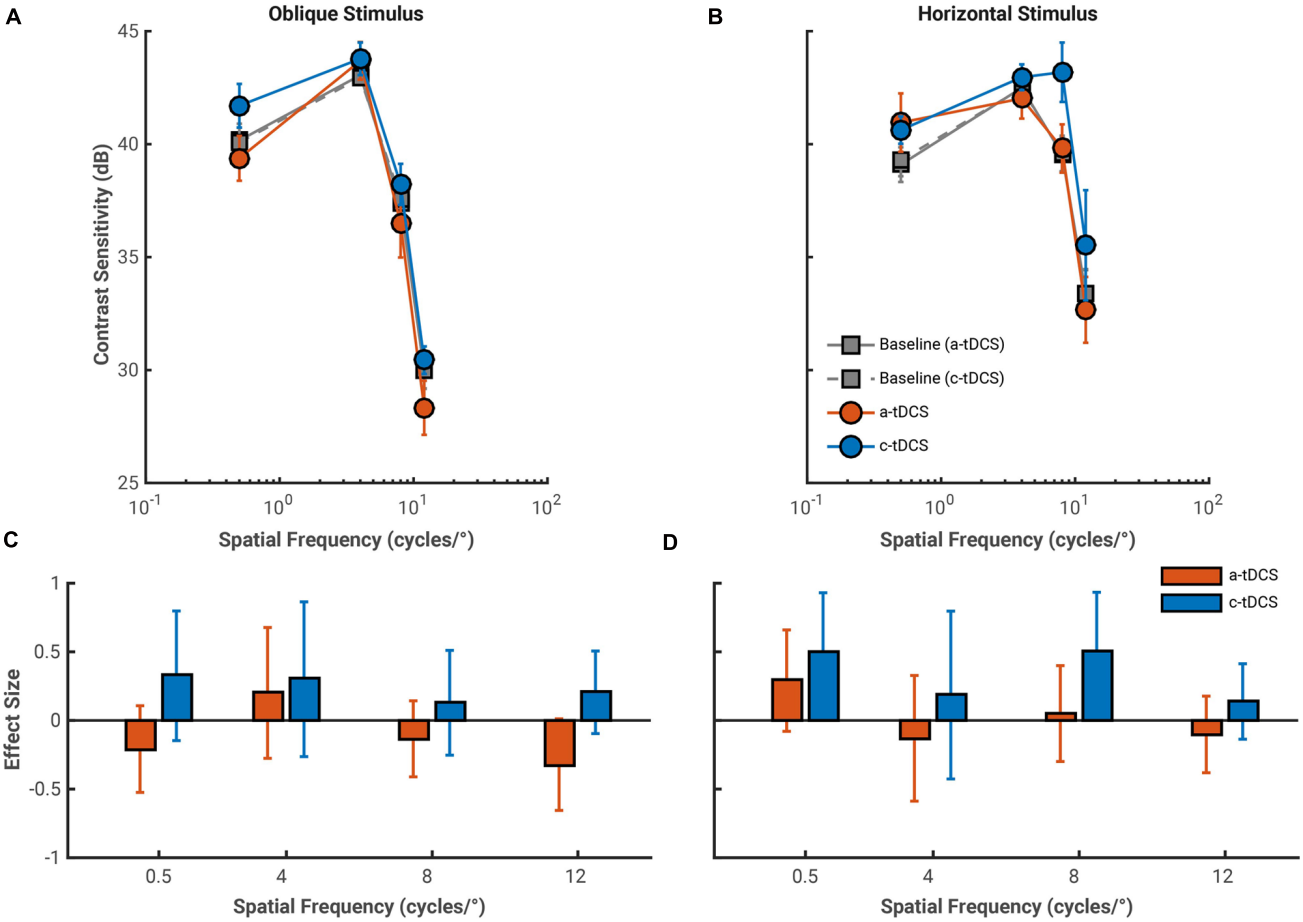
**Average pre-stimulation (gray) and stimulation CSFs for both a-tDCS (red) and c-tDCS (blue) measured with the oblique **(A)** and horizontal **(B)** fixed size gratings (at spatial frequencies of 0.5, 4, 8, and 12 cycles/°).** Contrast sensitivity is presented in decibels (dB). Error bars represent the standard error of the mean difference calculated across observers. **(C,D)** The effect sizes of the mean difference contrast sensitivity measured at stimulation and at pre-stimulation for oblique and horizontal conditions, respectively. We found a large increase in contrast sensitivity measured with the 8 cycles/° horizontal, fixed size grating under c-tDCS, and a potential polarity specific effect of tDCS on contrast sensitivity measured to oblique gratings at a spatial frequency of 12 cycles/°. Error bars represent the exact 95% confidence interval of the effect size. As in figure, we used error bar overlap to assess the magnitude of tDCS effects on contrast sensitivity.

The average effects of both a-tDCS and c-tDCS on oblique gratings of a fixed size are shown in **Figure 4**. There were no statistically significant interactions between spatial frequency and tDCS polarity for contrast sensitivity measure with either oblique, *F*(3,27) = 0.65, *p* = 0.585, $\eta_{p}^{2}$ = 0.068, or horizontal, *F*(3,27) = 2.83, *p* = 0.057, $\eta_{p}^{2}$ = 0.239, gratings. There was a main effect of tDCS polarity on contrast sensitivity measured to oblique gratings, *F*(1,9) = 9.23, *p* = 0.014, $\eta_{p}^{2}$ = 0.506. Anodal tDCS decreased and c-tDCS increased contrast sensitivity for all spatial frequencies. Effects of tDCS collapsed across spatial frequency are not particularly informative, and thus, we turn to our effect size analysis to measure if any changes in contrast sensitivity can attributed to tDCS.

Overall, effect sizes in the fixed size condition were small and had large confidence intervals. There is an indication of a polarity specific effect of tDCS on contrast sensitivity measured to an oblique grating at 12 cycles/°. This effect has a similar direction to the polarity specific effect obtain in the fixed period condition: a-tDCS decreased contrast sensitivity (*g* = –0.33, 95% CI [–0.65 0.01]) while c-tDCS increased sensitivity (*g* = 0.21, 95% CI [–0.10 0.51]). The influence of a-tDCS here does not seem to increase with spatial frequency. LTRs were similar for both 4 and 8 cycles/° conditions, and decreased slightly at 12 cycles/°, which suggest a narrowing of the contrast sensitivity distribution of a-tDCS (see **Table 2**). We found no meaningful effects of a-tDCS on contrast sensitivity measured with horizontal gratings, but did find an abnormal increase in contrast sensitivity under c-tDCS to a horizontal grating of 8 cycles/° (*g* = 0.51, 95% CI [0.06 0.93]). While this may be indicative of an actual facilitation in contrast sensitivity, the effects of c-tDCS in this stimulus condition seem independent of spatial frequency. Additionally, the LTR value for this condition was small in comparison to the magnitude of the effect size, which should be considered when interpreting this result.

**Table 2:** Left-Tail Ratios of contrast sensitivity measures in the fixed stimulus size condition.

		Spatial frequency (cycles/°)			
Stimulus dimensions		0.5	4	8	12
**45° Oblique**					
	a-tDCS	4.60	36.28	34.84	14.25
	c-tDCS	1.04	15.65	1.29^∗^	2.37^∗^
**Horizontal**					
	a-tDCS	2.07	109.22	2.61	4.17
	c-tDCS	4.70^∗^	1.05	1.60^∗^	26.57

*Values marked with an asterisk (^∗^) are ratios with the proportion of scores from the pre-stimulation distribution as the numerator.*

### Orientation Dependent Effects of tDCS

**FIGURE 5:**
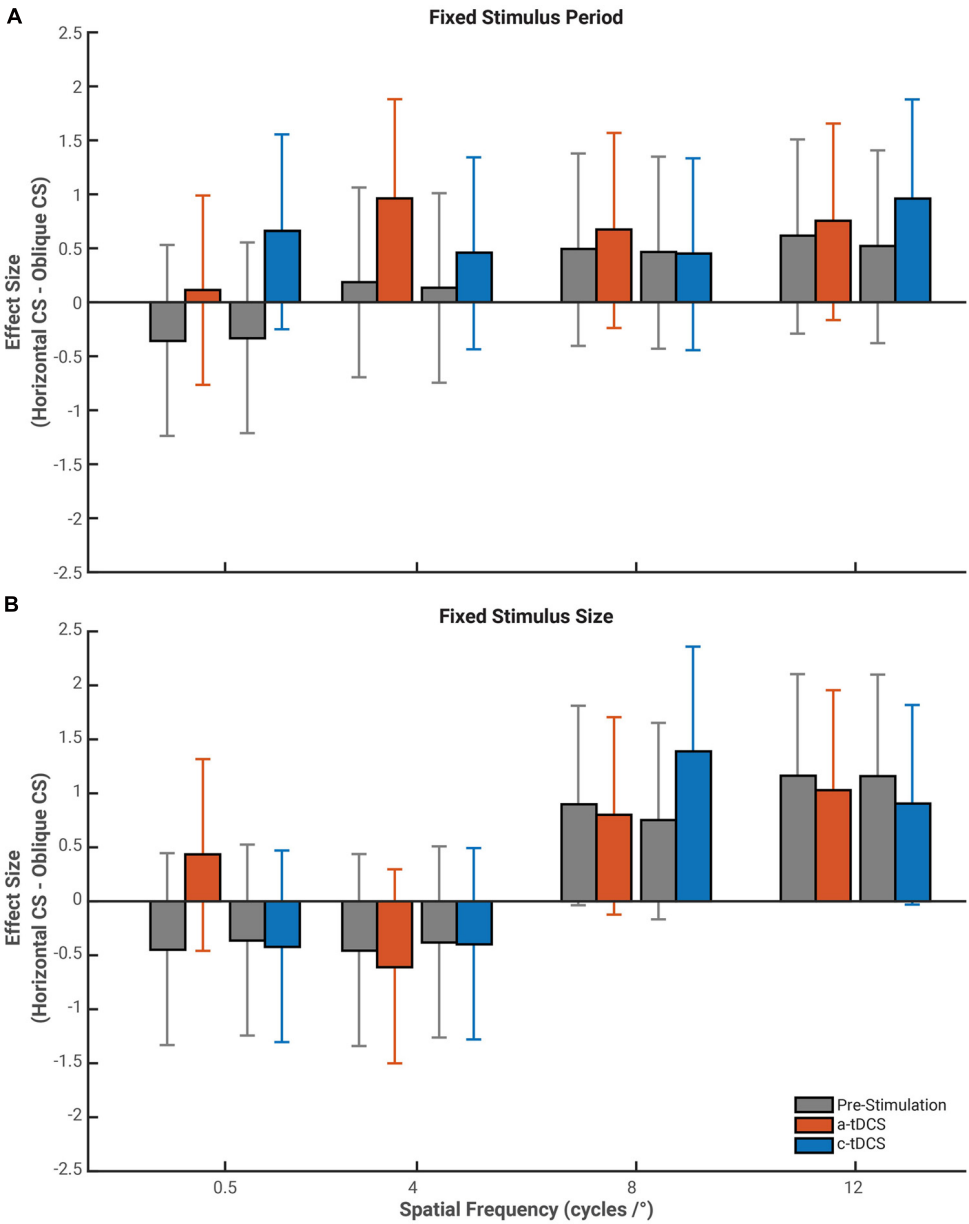
**Effect size of the mean difference between contrast sensitivity measured with horizontally orientated gratings and oblique orientated gratings.** Gray bars represent the respective pre-stimulation baseline for either a-tDCS (red) or c-tDCS (blue) contrast sensitivity difference between horizontal and oblique gratings for stimuli of a fixed period **(A)** and fixed size **(B)**. We do find a-tDCS to increase the difference between contrast sensitivity measured to horizontal gratings and that of oblique gratings at a spatial frequency of 4 cycles/° and for c-tDCS to have a similar effect at a spatial frequency of 12 cycles/°. Error bars represent the exact 95% confidence interval for the mean difference effect size.

Given that the effects of tDCS reported above varied according to the orientation of the stimulus, we opted compared the these effects directly by calculating effect size measures for the difference in contrast sensitivity between horizontal and oblique gratings for all stimulus and stimulation conditions (see **Figure 5**). Baseline contrast sensitivity, in both stimulus size conditions followed the well-defined “Oblique Effect” (Campbell et al., 1966; Appelle, 1972). Horizontal contrast sensitivity exceeded that of oblique at higher spatial frequencies in the fixed period (8 cycles/°: 12 cycles/°: *g* = 0.62, 95% CI [–0.29 1.51]) and fixed size conditions (8 cycles/°: *g* = 0.90, 95% CI [–0.04 1.81]; 12 cycles/°: *g* = 1.16, 95% CI [0.20 2.10]). However, the overlap between confidence intervals for baseline and tDCS suggest tDCS had no measurable impact on the magnitude of the Oblique Effect. Thus, while the effects of tDCS are orientation dependent (as shown above), they do not influence contrast sensitivity sufficiently to diminish or increase the magnitude of the Oblique Effect.

### Effects of tDCS on Low Spatial Frequency Contrast Sensitivity

Finally, we note that while contrast sensitivity to a grating with a spatial frequency of 0.5 cycles/° can be affected by tDCS, these effects are unlikely to be indicative of a true modulation. The 0.5 cycles/° grating were identical in both the fixed period and fixed size condition, and attributing contrast sensitivity to either condition was arbitrary in our analysis. When contrast sensitivity values from both stimulus size conditions (fixed period and fixed size) were combined, and the effects of tDCS reanalyzed, we find that both a-tDCS (*g* = 0.46, 95% CI [0.05 0.85], LTR = 1.61^∗^) and c-tDCS (*g* = 0.44, 95% CI [0.02 0.85], LTR = 4.44^∗^) increased contrast sensitivity from baseline equally. As both a-tDCS and c-tDCS had an identical influence on contrast sensitivity values, neither can serve as a control for the other, which clouds any meaningful effects we may have obtained at lower spatial frequencies. We had not anticipated any modulation of contrast sensitivity under tDCS for our lowest spatial frequency grating as it differed from all others used in this study. At 0.5 cycles/°, a grating is part of the low spatial frequency rollover in the CSF, and is presumably subject to additional inhibition than the other gratings (Webster and Miyahara, 1997; Meese and Hess, 2004). If the application of tDCS over primary visual cortex creates an imbalance in the interactive properties of neurons (i.e., excitatory and inhibitory interactions), regardless of polarity, then contrast sensitivity to low spatial frequency gratings may be affected differently by the current generated with tDCS than to high spatial frequencies. Our findings here suggest that the application of a current, regardless of polarity, will increase contrast sensitivity to low spatial frequencies. Why this is, however, remains unclear.

## Discussion

The goal of the current study was to assess whether the stimulus dimensions of gratings (spatial frequency, and orientation) could modulate the influence of tDCS on contrast sensitivity. We observe that the effects of both a-tDCS and c-tDCS were most pronounced on contrast sensitivity to obliquely oriented gratings of higher spatial frequency (i.e., above the peak of the CSF), and were absent at spatial frequencies below the peak the CSF. Generally, we found that a-tDCS decreased contrast sensitivity, while c-tDCS increased contrast sensitivity. However, these effects were small, and varied greatly across both stimulus spatial frequency, orientation and size conditions. In all but one stimulus condition, we found the influences of tDCS to be selective for polarity; only a-tDCS or c-tDCS had a large enough effect to influence contrast sensitivity. That said, when measured with an 8 cycles/° oblique grating (fixed period condition), contrast sensitivity was affected differently according to tDCS polarity: a-tDCS decreased while c-tDCS increased contrast sensitivity. Thus, while polarity specific effects of tDCS may be uncommon in vision studies (Antal et al., 2001; Accornero et al., 2007; Lang et al., 2007; Chaieb et al., 2008; Spiegel et al., 2012; Peters et al., 2013; Pirulli et al., 2014), we found that polarity specific influences of tDCS can be obtained under certain stimulus conditions (e.g., high frequency oblique gratings with small periods). Moreover, the effects of a-tDCS and c-tDCS on contrast sensitivity measured with fixed period gratings seem tied to orientation. Contrast sensitivity measured with oblique gratings was most subject to the influence of a-tDCS, while contrast sensitivity measured with horizontal gratings was most influenced by c-tDCS. While this did not affect the magnitude of the “Oblique Effect” (Campbell et al., 1966; Appelle, 1972; Essock, 1980), it may be indicative of an anisotropy of tDCS effects in vision, similar to the reported effects of Hansen et al. (2015).

The behavioral effects of tDCS result from an interaction between the electrical components of stimulation (Miranda et al., 2006; Paulus, 2011), the neuroanatomy of the stimulated area (Shipp, 2005; Radman et al., 2009; Bikson et al., 2013), the task completed by observers (Lapenta et al., 2013), and their cognitive state (Miniussi et al., 2010). While this allows for the broad acting effects of tDCS on cortex to be narrowed, or guided by the task, it also emphasizes that stimulus design should take into consideration the cortical area stimulated by tDCS. In primary visual cortex, the superficial layers near the apex of the calcarine sulcus contain neurons with higher preferred spatial frequencies than cells further from the apex (Tootell et al., 1981, 1988; De Valois et al., 1982; Foster et al., 1985; Engel et al., 1997; Horton, 2006; Henriksson et al., 2008; Yu et al., 2010). Additionally, the magnitude of the electric field generated by tDCS is greater at the cortical surface (Miranda et al., 2006; Nitsche et al., 2007; Bikson et al., 2013). Thus, it is plausible the effects of tDCS on contrast sensitivity were greatest when higher spatial frequency gratings were used as neurons with higher preferred spatial frequencies would be most influenced by tDCS. Likewise, the peak in current density at the apex of the primary visual cortex suggest the effects of tDCS may be restricted to the central visual field, which is retinotopically mapped to the apex of the calcarine sulcus (Tootell et al., 1988; Engel et al., 1997; Grill-Spector and Malach, 2004; Horton, 2006). There is a study that corroborates this hypothesis (Kraft et al., 2010), however, other factors may influence the localization of tDCS effects in the visual field, as a recent study by Costa et al. (2015) has failed to replicate the findings of Kraft et al. (2010). Nevertheless, if the effects of tDCS are greatest within the central 2° of the visual field, as proposed by Kraft et al., (2010), it may explain why contrast sensitivity to fixed size gratings, which extend beyond the area affected by tDCS, was only mildly altered by tDCS. Additional psychophysical mechanisms (e.g., summation effects; Graham et al., 1978; Legge, 1978; Peli et al., 1993; Meese and Summers, 2007) may have contributed to the lack of tDCS influence on contrast sensitivity to large gratings of high spatial frequency, as they also raise contrast sensitivity and potentially restricts any measurable influence of tDCS.

Changes in the stimulus characteristics presented to observers can have large contrasting tDCS effects on the same psychophysical measure. We opted to represent this with effect sizes to characterize changes in central tendency, and LTR, to define changes in the tail of the distribution (Feingold, 1995). While these may be considered uncommon statistical approaches, they are ideally suited to infer the meaningfulness of a change in behavior attributed to tDCS. For example, effects of tDCS in the tails of a distribution are to be expected as not all observers are affected equally by tDCS (Wagner et al., 2007; Datta et al., 2009; Spiegel et al., 2013). Thus, we used LTR to better define our dataset and characterized not only average effects (group-level) but also account for individual differences. Our analyses demonstrated that while the changes in contrast sensitivity induced by tDCS were sufficiently large to shift the central tendency of a distribution, certain effects were most apparent in the tails of the distribution. The decrease in contrast sensitivity under a-tDCS to fixed period gratings was of a similar magnitude for spatial frequencies of 4, 8, and 12 cycles/°, but the proportion of contrast sensitivity values in the left tail of the distribution increased with spatial frequency. This suggests observer contrast sensitivity, generally, was much more likely to show an influence of a-tDCS in higher spatial frequency conditions than when the spatial frequency neared the peak of the CSF. Furthermore, we calculated 95% confidence intervals of effect size measures to obtain an estimate of the sampling error in our effects. While most effect sizes were of moderate size, many had large confidence intervals that contained both positive and negative values. As 95% of all confidence intervals calculated in this way will contain the true effect size of a-tDCS and c-tDCS on contrast sensitivity measurements, both increments and decrements in contrast sensitivity appear equally valid directions in many conditions evaluated here. Hence, the expected directionality of tDCS polarity – a-tDCS excites while c-tDCS inhibits – which stems predominantly from findings in motor cortex (Nitsche et al., 2003a, 2007; Stagg et al., 2009; Jacobson et al., 2012; Pellicciari et al., 2013), should be disregarded for cortical areas that are functionally and structurally different (Shipp, 2005, 2007).

### tDCS Polarity and Psychophysical Performance

We found facilitatory and inhibitory effects of tDCS on low-level visual function, but our findings contrast those of other, similar studies (Antal et al., 2001; Chaieb et al., 2008; Kraft et al., 2010; Peters et al., 2013; Spiegel et al., 2013). It well established that the a-tDCS excitatory, c-tDCS inhibitory effect is only truly valid when measured in motor cortex, while in visual cortex the behavioral outcome of tDCS cannot necessarily be predicted by its polarity (Antal et al., 2004a; Accornero et al., 2007; Miniussi et al., 2013; Pirulli et al., 2014; Hansen et al., 2015). There are many factors that contribute to the net influence of current on cell activity that may explain the different outcomes between stimulation in motor and primary visual cortex (e.g., neuroanatomy and functional anatomy; Radman et al., 2009; Peterchev et al., 2012; Bikson et al., 2013; Rahman et al., 2013). Still, if cells in primary cortex are similarly influenced by tDCS as those of motor cortex, an additional mechanism must be defined to account for the variability in behavioral outcomes of tDCS in vision studies. For tasks that involve the detection of a stimulus, facilitatory effects of c-tDCS may stem from an increase in signal-to-noise ratios that result from a decrease in cell excitability (Antal et al., 2004b; Miniussi et al., 2013; Pirulli et al., 2014). An increase in the signal-to-noise ratio could minimize stimulus uncertainty (Pelli, 1985), which will increase the detectability of the stimulus. Similarly, a-tDCS could worsen performance by injecting additional noise and decreasing the signal-to-noise ratio. That said, tDCS is a continuous neurostimulation procedure and its effects on neuronal behavior cannot be as simple as an increment in excitability under a-tDCS and decrement in excitability under c-tDCS (Miniussi et al., 2013; Pirulli et al., 2014). The continuous current generated by tDCS may instead alter the balance of excitation and inhibition in neurons affected by the current (Pirulli et al., 2014). Balance of excitation and inhibition is a known neuro-mechanism responsible for the tuning characteristics of visually responsive cells (it serves to narrow the bandwidth of tuning curves and regulates their responses to contrast; Rose and Blakemore, 1974; Blin et al., 1993; Ferster and Miller, 2000; Li et al., 2008; Edden et al., 2009; Katzner et al., 2011). Thus, the psychophysical performance change under tDCS obtained in vision studies, such as the one presented here, may lie in low-level gain mechanisms that adjust the responses of a cell to a given level of contrast.

### Limitations

Our tDCS stimulation protocol used large electrodes (48 cm^2^ over Oz and 96 cm^2^ over Cz), which most likely covered both primary visual and secondary visual cortical areas. As these areas differ in their cortical folding (Rosa et al., 1997a,b; Horton, 2006), the alignment between the current generated by tDCS to the somatodendritic axis of the cell will vary and potentially alter the polarizing effects of tDCS (Rushton, 1927; Radman et al., 2009; Rahman et al., 2013). It is unclear how the stimulation of both primary and secondary visual cortex may have impacted our findings here, however, more focal approaches that use smaller electrodes (HD-tDCS; Miranda et al., 2013; Rahman et al., 2013), may help prevent the simultaneous stimulation of multiple visually responsive cortical sites in future studies.

## Conclusion

The effects of tDCS on contrast sensitivity are largest when measured with high spatial frequency oblique oriented gratings of a fixed period (1.5 cycles). Additionally, we found that the magnitude of a-tDCS and c-tDCS effects may be anisotropic, as c-tDCS generally elicited larger effects with horizontal gratings, while a-tDCS with oblique gratings. Finally, the overall magnitude of tDCS effects on contrast sensitivity were small, and spatial frequency dependent effects vanished when contrast sensitivity was measured with larger gratings of variable period. The effects of tDCS on low-level visual function is evidently subject to the particular stimulus attributes presented to observers, and further demonstrates the susceptability of this stimulation technique to the activity of cells within the cortical area it stimulates. In regards to contrast sensitivity, we find that under certain stimulus condition, tDCS effects may be facilitatory or inhibitory within a particular group of observers, regardless of stimulation polarity. Consequently, careful use of stimuli that reliably elicit tDCS polarity specific effects should be favored when implementing tDCS in vision studies.

## Conflict of Interest Statement

The authors declare that the research was conducted in the absence of any commercial or financial relationships that could be construed as a potential conflict of interest.

## Footnotes

1 The contrast sensitivity function is an index of sensitivity to contrast across multiple spatial frequencies, but previous studies that have used contrast sensitivity as a dependent measure of tDCS have restricted their stimulus to a single spatial frequency, orientation, and size. This can alter contrast sensitivity and thus alter tDCS effects. For example, CSFs measured with full-field gratings (well localized in Fourier space) generally have narrower bandpass shape and peak at a higher spatial frequency (∼4 cycles/°) than CSFs measured with gratings localized both in spatial frequency and space (i.e., Gabors), which peak at about 1 cycle/° (Peli et al., 1993). Similarly, CSFs measured with cardinally oriented gratings have higher contrast sensitivity values at spatial frequencies above the peak of the CSF than when measured with obliquely oriented gratings (Campbell et al., 1966).

2 We note that these results stem from a single study, which has yet to have been replicated.
