# Supplementary material for: The Effects of tDCS Across the Spatial Frequencies and Orientations that Comprise the Contrast Sensitivity Function
Source: Front Psychol. 2015 Nov 27;6:1784. doi: 10.3389/fpsyg.2015.01784 (PMC4661264; doi:10.3389/fpsyg.2015.01784)
Supplement: Supplementary file 1 [file Data_Sheet_1.DOCX]

**Supplementary Material A**

The effects of tDCS across the Spatial Frequencies and Orientations that comprise the Contrast Sensitivity Function

Bruno Richard^1,2^, Aaron P. Johnson^1^, Benjamin Thompson^3,4^, Bruce C. Hansen^5^

^1^ Department of Psychology, Concordia University, Montreal, Quebec, Canada

^2^ Department of Psychology, University of York, Heslington, York, United Kingdom

^3^School of Optometry, and Vision Science, University of Waterloo, Ontario, Canada

^4^School of Optometry and Vision Science, The University of Auckland

^5^Department of Psychology and Neuroscience Program, Colgate University, Hamilton, New York, USA

Correspondence to Bruno Richard, Department of Psychology, University of York, Heslington, York, YO10 5DD

Office: +44 1904 322879

Email: bruno.richard@york.ac.uk

Baseline Sequential Measurement in Time Data

The baseline portion of this study, completed by all observers, measured their contrast sensitivity to each spatial frequency by size stimulus blocks 10 times. The first two repetitions were practice staircases and removed prior to data analysis. Contrast sensitivity values for all observers in this study (both 45° oblique and horizontal orientation groups) for the final 8 staircases completed during baseline are shown in Figure A1. As described in text, we calculated the linear regression line of best fit for all observers across the 8 sequential measurements in time for all stimulus dimensions (solid lines in Figure A1) and found that no slope deviated from 0. Therefore, contrast sensitivity value for all 20 observers remained relatively stable across the final 8 repetitions of baseline measurements.

We opted to combine both the contrast sensitivity measured during baseline and the pre-stimulation contrast sensitivity measures to use as a pre-stimulation baseline in our data analyses reported in text. There is evidence that same-day and different-day baseline measures may alter the relative effects of tDCS (Peters et al., 2013), however, as observers were constrained to perform similarly to their baseline contrast sensitivity measurements prior to undergoing stimulation, we found no differences in our effects when using either different-day baseline measures or same-day baseline measures alone or combined (see **Figure A2**).

Figure A1. Observers in the 45° oblique grating group and horizontal grating group showed no statistically significant difference across the sequential measurements in time for all stimulus dimensions used in this study (all *p*s > 0.05). Each color in the figure represents the contrast sensitivity value for an individual observer for the final 8 measurements of contrast sensitivity (in decibels) completed in the baseline portion of the study.

**Figure A2.** Average contrast sensitivity values (n = 10 observers per data point) for the last 8 spatial frequency by stimulus size blocks (fixed period and fixed size) of the baseline session, and the pre-stimulation contrast sensitivity values for both for a-tDCS (red) and c-tDCS (blue) sessions. The figure is split by stimulus orientations. Average contrast sensitivity values did not change significantly between baseline measurements sessions or pre-stimulations. Error bars represent 1 standard error of the mean (note that many data points have error bars smaller than the marker).

**References**

Peters, M. A. K., Thompson, B., Merabet, L. B., Wu, A. D., and Shams, L. (2013). Anodal tDCS to V1 blocks visual perceptual learning consolidation. *Neuropsychologia* 51, 1234–1239. doi:10.1016/j.neuropsychologia.2013.03.013.
