# Supplementary material for: The Effects of tDCS Across the Spatial Frequencies and Orientations that Comprise the Contrast Sensitivity Function
Source: Front Psychol. 2015 Nov 27;6:1784. doi: 10.3389/fpsyg.2015.01784 (PMC4661264; doi:10.3389/fpsyg.2015.01784)
Supplement: Supplementary file 2 [file Data_Sheet_2.DOCX]

**Supplementary Material B**

The effects of tDCS across the Spatial Frequencies and Orientations that comprise the Contrast Sensitivity Function

Bruno Richard^1,2^, Aaron P. Johnson^1^, Benjamin Thompson^3,4^, Bruce C. Hansen^5^

^1^ Department of Psychology, Concordia University, Montreal, Quebec, Canada

^2^ Department of Psychology, University of York, Heslington, York, United Kingdom

^3^School of Optometry, and Vision Science, University of Waterloo, Ontario, Canada

^4^School of Optometry and Vision Science, The University of Auckland

^5^Department of Psychology and Neuroscience Program, Colgate University, Hamilton, New York, USA

Correspondence to Bruno Richard, Department of Psychology, University of York, Heslington, York, YO10 5DD

Office: +44 1904 322879

Email: bruno.richard@york.ac.uk

**Description of Statistical Analyses**

This appendix offers a brief description of analyses used in this study to estimate the magnitude of both a-tDCS and c-tDCS effects on contrast sensitivity. We begin with a complete table of U1 statistics used to assess overlap between the Colgate University and Concordia Samples, and subsequently offer an overview of building exact [(1-α)*100] confidence intervals around the Hedge’s g effect size, and define the computation of Left Tail Ratios (LTRs). Further details on these analyses and calculations can be found in Kline (2004) and Cumming and Finch (2001). Finally, we report the output of all repeated-measures ANOVA from our experiment.

**Measures of overlap**

Given the large discrepancy in sample size between the Colgate (n = 8) and Concordia (n = 2) samples, we opted to assess overlap between the two prior to averaging their data with a simple measure of overlap, U1, which defines the proportion of scores between two sampling distribution that do not overlap (Cohen, 1988). U1 is calculated as follows: 1) count the total number of scores in one group outside the range of scores in the other group and 2) divide that number by total sample size (N). If the mean contrast between both groups is 0, then U1 is also 0, while it is 1 if both samples do not overlap whatsoever (see **Table B1**).

Table B1. Measures of overlap between the Colgate and Concordia samples (U1) for all stimulus block and time points (Baseline / tDCS) of the study.

|  | Baseline | | tDCS | |
| --- | --- | --- | --- | --- |
|  | a-tDCS | c-tDCS | a-tDCS | c-tDCS |
| Spatial Frequency | Fixed Stimulus Period | | | |
| 0.5 | 0.15 | 0.15 | 0.38 | 0.13 |
| 4 | 0.28 | 0.20 | 0.06 | 0.06 |
| 8 | 0.15 | 0.16 | 0.31 | 0.19 |
| 12 | 0.05 | 0.05 | 0.25 | 0.00 |
|  | Fixed Stimulus Size | | | |
| 0.5 | 0.05 | 0.05 | 0.06 | 0.19 |
| 4 | 0.16 | 0.13 | 0.88 | 0.19 |
| 8 | 0.11 | 0.13 | 0.25 | 0.00 |
| 12 | 0.03 | 0.03 | 0.13 | 0.13 |

**Group-Level Analyses**

Traditionally, confidence intervals are constructed in order to estimate the mean of the sampling distribution of the parameter of interest (μ), as the mean of the sampling distribution will be within the confidence interval [(1-α)*100] percent of the time. There are, other approaches to build confidence intervals, which are more intuitive when building a confidence interval around an effect size measure. Instead of defining the confidence intervals as capturing μ [(1-α)*100] percent of the time, the confidence interval is built by defining plausible values of μ. Therefore, the lower limit of the confidence interval is defined as all plausible values of μ having a [(1- α/2)*100] probability below a certain value of x, while the upper limit is defined as all plausible values of μ having a [(α/2)*100] probability below x. In this form, the confidence intervals can be calculated by finding the mean of the distribution for which [(1-α/2)*100] of its proportion lies below the effect size measure (the lower limit) and the mean of a distribution for which [(α/2)*100] of its proportion falls below the effect measure (the upper limit; see **Figure B1**).

The sampling distribution of effect sizes (g) is a non-central *t* distribution: a probability density function defined by two parameters, the degrees of freedom (*df*) and a non-centrality parameter (Δ). The non-centrality parameter reflects the degree to which the null hypothesis is false. If Δ = 0, the resulting distribution is a symmetrical central *t* distribution with the same *df*, while it will be positively skewed when Δ > 0 and negatively skewed when Δ < 0. In an independent samples design, the effect size between two sample means is related to the non-centrality parameter as follows:

|  |  | **Equation B1** |
| --- | --- | --- |

When building confidence intervals around an effect size for a dependent samples design, as we have done here, exact confidence intervals can only be defined when the mean difference is standardize by the standard deviation of the difference scores (s_D_). Effect sizes standardized by the within-group pooled standard deviation or by the standard deviation of a single group are too complex and do not follow a central or non-central *t* distribution. In a dependent samples design, the effect size is related to the non-centrality parameter as follows:

|  |  | **Equation B2** |
| --- | --- | --- |

Whereby the variance of the difference scores () is defined as

|  |  | **Equation B3** |
| --- | --- | --- |

where cov_12_ is the covariance of the observed scores across conditions and is the product of the cross condition correlation and the within condition standard deviations.

As both the non-centrality parameter and effect size are linked, we can build confidence intervals around an effect size measure by first building a confidence interval for the non-centrality parameter and then transforming it into the effect size units. For a given *t* statistic (independent: ; dependent: $t=M_{D}/s_{m_{D}}$), we can search for the non-central *t* distribution with Δ such that [1- α/2]*100 falls below the *t* statistic (the lower limit, Δ_L_) and conversely find the non-central *t* distribution with Δ such that [α/2]*100 falls below *t* (the upper limit, Δ_U_). **Figure B1** illustrates this concept for an independent sample design with values taken from Kline (2004). When both the Δ_L_ and Δ_U_ have been found, they can easily be converted into effect size values (**equation B1** or **equation B2**).

Finding the appropriate non-central *t* distribution is simple in MATLAB, as the non-central *t* distribution is defined by the *nctcdf* function (requires the statistics toolbox, see attached MATLAB code). Statistical software, including SAS and STATISTICA also include non-central *t* distribution calculators that allow building exact confidence intervals around an effect size, and the Real Statistics Excel Resource Pack (http://www.real-statistics.com/) also contains a non-central *t* distribution calculator. We strongly encourage the construction of confidence intervals around effect sizes, as the effect, just as any other statistics, will always be subject to estimation error. Estimated confidence intervals for effect sizes can be calculated with more traditional (estimating the mean of the sampling distribution that will capture a value [(1-α/2) *100] percent of the time) by using the z distribution (see **Equation B4**).

|  | $\begin{matrix} g\pm z_{\alpha/2}s_{d} \\ s_{d}=\sqrt{\frac{g^{2}}{2df}+\frac{1}{n_{1}}+\frac{1}{n_{2}}} \end{matrix}$ | **Equation B4** |
| --- | --- | --- |

**Figure B1.** Finding the two non-central *t* distributions with best fitting non-centrality parameters (Δ_L_ and Δ_U_). The blue *pdf* is the non-central t distribution with the best fitting Δ_L_ for which a cumulative density of [1-α/2]*100 falls below *t*. The orange distribution is the best fitting Δ_U_ for which a cumulative density of [α/2]*100 falls below *t*. Values taken from Kline (2004).

**Case-Level Analyses**

Transcranial Direct Current Stimulation in human observers are subject to a variety of factors that include the skull density, and alignment of cortical gyri with the electrodes that will moderate the effects of stimulation and vary significantly between observers (Miranda et al., 2006; Datta et al., 2009; Sadleir et al., 2010). Furthermore, given the magnitude of the non-shunted direct current that enters cortex is several orders of magnitude less than what is required to elicit action potentials (Rahman et al., 2013; Peterchev et al., 2012; Creutzfeldt et al., 1962), we expected contrast sensitivity values obtain during stimulation of have different variance than those obtain prior to stimulation (as some observers may respond more drastically than others to stimulation). Previous attempts to account for individual variability between observers receiving tDCS have predominantly focused on the removal of “non-responders” (observers that shown small or effects in the opposite direction typically reported for a tDCS polarity), we opted to implement case-level analyses, which allowed us to keep all observers that underwent tDCS in our data analysis. Case-level analyses can be particularly beneficial when the variance between two samples (here pre-stimulation and stimulation) is believed to differ significantly, but their central tendency may not (see **Figure B2)**. Case-level analyses are therefore ideally suited to quantify the effects of neuro-modulators, including tDCS, as the effects of stimulation are known to be small (Jacobson et al., 2012) at the level of central tendency, but may induce large effects in certain observers more susceptible to neuro-stimulation. There are many forms of case-level analyses (see Kline, 2004), however, given that our data showed large suppressive effects of a-tDCS, we opted to measure the Left-Tail Ratios for all stimulus dimensions presented in the results section.


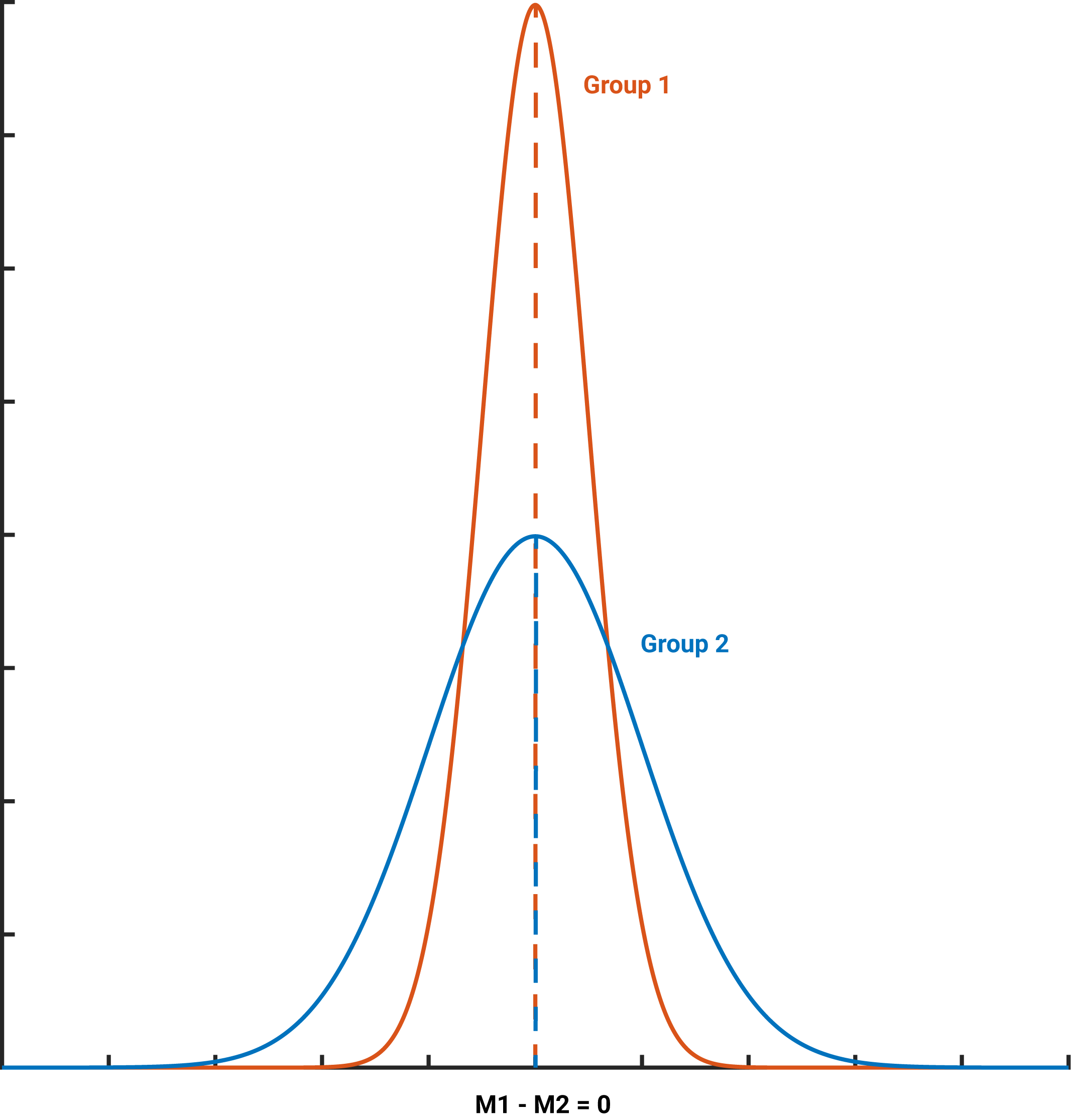


**Figure B2**. In this scenario, two sample distributions have identical means, but different variance values. The effect size of the mean difference value here is 0, however, this does not mean that these two distributions are identical. LTRs, which calculate the proportion of scores with the tails of a combined distribution (the “average” distribution of both samples) would identify the difference between these two samples. The combination of both effect size measures and tail ratios ensures that any effect that leads to a change in the sample distribution is properly characterized.

A Left-tail ratio is the relative proportion of scores from two different groups (here contrast sensitivity collected prior to and during tDCS) that fall in the lower extreme (left-tail) of the combined frequency distribution. Tail ratios are always calculated with the largest value as the numerator and are therefore always larger than 1. We computed left-tail ratios based on a cut-off point relative to the mean (*M_t_*) and standard deviation (*s_T_*) of the combined distribution (pre-stimulation and stimulation) for each stimulus dimension presented to observers in this study. The mean and standard deviation of the combined distribution are calculated as follows.

|  |  | **Equation B4** |
| --- | --- | --- |

The cut-point for left tail ratios is defined as one standard deviation below the grand mean. The distance between the cut-off score (*M_T_ – s_T_*) and the mean of each sample is then converted into a z-score and the proportion of scores that fall below this z-score under the normal distribution is calculated. The left-tail ratio is then simply the ratio between both proportions, with the largest proportion always placed as the numerator.

|  |  | **Equation B5** |
| --- | --- | --- |

**Repeated Measures ANOVA Tables**

**Table B2 –** Repeated Measures ANOVA – Fixed Period Stimulus Conditions

| Factors | SS | *df* | MS | F | *p* | $\eta_{p}^{2}$ |
| --- | --- | --- | --- | --- | --- | --- |
| Oblique | | | | | | |
| tDCS | 1.98 | 1 | 1.98 | 0.52 | 0.489 | 0.055 |
| SF | 5.19 | 3 | 1.73 | 0.29 | 0.830 | 0.032 |
| tDCS x SF | 72.30 | 3 | 24.10 | 8.10 | 0.001 | 0.474 |
| Subjects | 57.57 | 9 | 6.40 |  |  |  |
| tDCS x Subjects | 34.17 | 9 | 3.80 |  |  |  |
| SF x Subjects | 159.36 | 27 | 5.90 |  |  |  |
| tDCS x SF x Subjects | 80.29 | 27 | 2.97 |  |  |  |
| Total | 410.85 | 79 | 5.20 |  |  |  |
| Horizontal | | | | | | |
| tDCS | 12.27 | 1 | 12.27 | 1.08 | 0.325 | 0.107 |
| SF | 14.26 | 3 | 4.75 | 0.58 | 0.631 | 0.061 |
| tDCS x SF | 39.13 | 3 | 13.04 | 1.97 | 0.142 | 0.179 |
| Subject | 284.48 | 9 | 31.61 |  |  |  |
| tDCS x Subject | 101.86 | 9 | 11.32 |  |  |  |
| SF x Subject | 219.68 | 27 | 8.14 |  |  |  |
| tDCS x SF x Subject | 178.88 | 27 | 6.63 |  |  |  |
| Total | 850.56 | 79 | 10.77 |  |  |  |

**Table B3 –** Simple Effect Comparison - Fixed Period Oblique Stimuli

| Factors | SS | *df* | MS | F | *p* | $\eta_{p}^{2}$ |
| --- | --- | --- | --- | --- | --- | --- |
| tDCS _at SF = 0.5 cpd_ | 39.90 | 1 | 39.90 | 4.72 | .058 | .344 |
| Subjects | 98.72 | 9 | 10.97 |  |  |  |
| tDCS X Subjects | 76.06 | 9 | 8.45 |  |  |  |
| Total | 214.67 | 19 | 11.30 |  |  |  |
|  |  |  |  |  |  |  |
| tDCS _at SF = 4 cpd_ | 3.34 | 1 | 3.34 | 1.57 | .242 | .149 |
| Subjects | 36.60 | 9 | 4.07 |  |  |  |
| tDCS X Subjects | 19.12 | 9 | 2.12 |  |  |  |
| Total | 59.06 | 19 | 3.11 |  |  |  |
|  |  |  |  |  |  |  |
| tDCS _at SF = 8 cpd_ | 26.35 | 1 | 26.35 | 20.79 | 0.001 | 0.698 |
| Subjects | 34.83 | 9 | 3.87 |  |  |  |
| tDCS X Subjects | 11.41 | 9 | 1.27 |  |  |  |
| Total | 72.58 | 19 | 3.82 |  |  |  |
|  |  |  |  |  |  |  |
| tDCS _at SF = 12 cpd_ | 4.70 | 1 | 4.70 | 5.37 | .046 | .374 |
| Subjects | 46.77 | 9 | 5.20 |  |  |  |
| tDCS X Subjects | 7.88 | 9 | 0.88 |  |  |  |
| Total | 59.35 | 19 | 3.12 |  |  |  |

**Table B4 –** Repeated Measures ANOVA – Fixed Size Stimulus Conditions

| Factors | SS | *df* | MS | F | *p* | $\eta_{p}^{2}$ |
| --- | --- | --- | --- | --- | --- | --- |
| Oblique | | | | | | |
| tDCS | 48.45 | 1 | 48.45 | 9.23 | 0.014 | 0.506 |
| SF | 20.46 | 3 | 6.82 | 1.35 | 0.278 | 0.131 |
| tDCS x SF | 14.41 | 3 | 4.80 | 0.66 | 0.585 | 0.068 |
| Subjects | 64.89 | 9 | 7.21 |  |  |  |
| tDCS x Subjects | 47.25 | 9 | 5.25 |  |  |  |
| SF x Subjects | 135.93 | 27 | 5.03 |  |  |  |
| tDCS x SF x Subjects | 197.21 | 27 | 7.30 |  |  |  |
| Total | 528.60 | 79 | 6.69 |  |  |  |
| Horizontal | | | | | | |
| tDCS | 56.38 | 1 | 56.38 | 4.80 | 0.056 | 0.348 |
| SF | 45.33 | 3 | 15.11 | 0.97 | 0.423 | 0.097 |
| tDCS x SF | 48.72 | 3 | 16.24 | 2.83 | 0.057 | 0.239 |
| Subject | 278.77 | 9 | 30.97 |  |  |  |
| tDCS x Subject | 105.70 | 9 | 11.74 |  |  |  |
| SF x Subject | 421.96 | 27 | 15.63 |  |  |  |
| tDCS x SF x Subject | 154.98 | 27 | 5.74 |  |  |  |
| Total | 1111.84 | 79 | 14.07 |  |  |  |

**References**

Cohen, J. (1988). *Statistical power analysis for the behavioral sciences*. 2nd ed. New Jersey: Lawrence Erlbaum Associates.

Creutzfeldt, O. D., Fromm, G. H., and Kapp, H. (1962). Influence of transcortical d-c currents on cortical neuronal activity. *Exp. Neurol.* 5, 436–452. doi:10.1016/0014-4886(62)90056-0.

Cumming, G., and Finch, S. (2001). A Primer on the Understanding, Use, and Calculation of Confidence Intervals that are Based on Central and Noncentral Distributions. *Educ. Psychol. Meas.* 61, 532–574. doi:10.1177/0013164401614002.

Datta, A., Bansal, V., Diaz, J., Patel, J., Reato, D., and Bikson, M. (2009). Gyri -precise head model of transcranial DC stimulation: Improved spatial focality using a ring electrode versus conventional rectangular pad. *Brain Stimul.* 2, 201–207. doi:10.1016/j.brs.2009.03.005.

Kline, R. B. (2004). *Beyond Significance Testing: Reforming Data Analysis Methods in Behavioral Research*. Washington: American Psychological Association.

Miranda, P. C., Lomarev, M., and Hallett, M. (2006). Modeling the current distribution during transcranial direct current stimulation. *Clin. Neurophysiol.* 117, 1623–1629. doi:10.1016/j.clinph.2006.04.009.

Peterchev, A. V., Wagner, T. a., Miranda, P. C., Nitsche, M. a., Paulus, W., Lisanby, S. H., Pascual-Leone, A., and Bikson, M. (2012). Fundamentals of transcranial electric and magnetic stimulation dose: definition, selection, and reporting practices. *Brain Stimul.* 5, 435–53. doi:10.1016/j.brs.2011.10.001.

Peters, M. A. K., Thompson, B., Merabet, L. B., Wu, A. D., and Shams, L. (2013). Anodal tDCS to V1 blocks visual perceptual learning consolidation. *Neuropsychologia* 51, 1234–1239. doi:10.1016/j.neuropsychologia.2013.03.013.

Rahman, A., Reato, D., Arlotti, M., Gasca, F., Datta, A., Parra, L. C., and Bikson, M. (2013). Cellular effects of acute direct current stimulation: somatic and synaptic terminal effects. *J. Physiol.* 591, 2563–78. doi:10.1113/jphysiol.2012.247171.

Sadleir, R. J., Vannorsdall, T. D., Schretlen, D. J., and Gordon, B. (2010). Transcranial direct current stimulation (tDCS) in a realistic head model. *Neuroimage* 51, 1310–1318. doi:10.1016/j.neuroimage.2010.03.052.
